# The relation between insulin resistance and lung function: a cross sectional study

**DOI:** 10.1186/s12890-015-0125-9

**Published:** 2015-11-06

**Authors:** Gul Sagun, Canan Gedik, Esra Ekiz, Engin Karagoz, Mumtaz Takir, Aytekin Oguz

**Affiliations:** Department of Internal Medicine, Istanbul Medeniyet University Goztepe Training and Research Hospital, Istanbul, Turkey; Department of Respiratory Disease, Istanbul Medeniyet University Goztepe Training and Research Hospital, Istanbul, Turkey; Department of Endocrinology, Istanbul Medeniyet University Goztepe Training and Research Hospital, Istanbul, Turkey

**Keywords:** Insulin resistance, Lung function, Metabolic syndrome, Obesity, Spirometry

## Abstract

**Background:**

Impaired lung function and insulin resistance have been associated and thereby have also been indicated to be powerful predictors of cardiovascular mortality. Therefore, the co-existence of insulin resistance and impaired lung function accompanied with cardiovascular risk factors should induce cardiovascular mortality even in patients without known respiratory disease in a cumulative pattern. It could be useful to determine the lung function of patients with insulin resistance in order to decrease cardiovascular mortality by means of taking measures that minimize the risk of decline in lung function. However, no prior studies have been done on association between insulin resistance and lung function in adults in Turkey. We aimed to determine if insulin resistance plays a detrimental role in lung function in outpatients admitted to internal medicine clinics in adults from Turkey.

**Methods:**

A total of 171 outpatients (mean ± SD) age: 43.1 ± 11.9) years) admitted to internal medicine clinics were included in this single-center cross-sectional study, and were divided into patients with (*n* = 63, mean ± SD) age: 43.2 ± 12.5) years, 83.5 % female) or without (*n* = 108, mean ± SD) age: 43.0 ± 11.6) years, 93.5 % female) insulin resistance. All patients were non-smokers. Data on gender, age, anthropometrics, blood pressure, blood biochemistry, metabolic syndrome (MetS), and lung function tests were collected in each patient. Correlates of insulin resistance were determined via logistic regression analysis.

**Results:**

Insulin resistance was present in 36.8 % of patients. Logistic regression analysis revealed an increase in the likelihood of having insulin resistance of 1.07 times with every 1-point increase in waist circumference, 1.01 times with every 1-point increase in triglycerides, 0.93 times with every 1-point decrease in HDL (high density lipoprotein) cholesterol, and 0.86 times with every 1-point decrease in percentage of FEV1/FVC _pre_ (FEV1_%pre_: Forced expiratory volume in the first second of expiration for predicted values; FVC_%pre_.: Forced vital capacity for predicted values).

**Conclusions:**

Insulin resistance should also be considered amongst the contributing factors for decline in lung function.

**Electronic supplementary material:**

The online version of this article (doi:10.1186/s12890-015-0125-9) contains supplementary material, which is available to authorized users.

## Background

Impaired lung function, as measured by forced vital capacity (FVC) or forced expiratory volume in the first second (FEV1) [1] has been indicated as not only a marker of premature death from all causes [2] but also has been associated with excess adiposity, insulin resistance, MetS, and type 2 diabetes mellitus. All these conditions have also been indicated to be powerful predictors of nonfatal ischemic heart disease and cardiovascular mortality [1, 3–6].

Insulin resistance, beta cell dysfunction, impaired glucose tolerance, and MetS ultimately lead to T2DM. In other words, insulin resistance has been associated with a range of cardiovascular risk factors including dyslipidemia, essential hypertension, glucose intolerance, and diabetes [7]. While reduced baseline FVC and FEV1 were reported to be independently related to a greater risk of future development of MetS as well as new onset type 2 diabetes mellitus [8], of which insulin resistance is a core factor. Diabetes mellitus has also been considered amongst the contributing factors for the development of obstructive lung disease [8] and associated with greater rates of decline in ventilatory function in longitudinal studies [4, 9]. However, the underlying mechanism is still unclear.

Although impairment of lung function has been reported to precede the development of diabetes [10, 11], studies concerning the association of lung dysfunction and hyperglycemia in individuals without diabetes, including impaired fasting plasma glucose (FPG) and elevated hemoglobin A1c (HbA1c) concentrations, revealed inconsistent results [10, 12–14]. While the exact mechanisms by which a diabetic state leads to low lung function and whether a low lung function is predictive of development of diabetes remains to be elucidated [10], obesity, chronic systemic inflammation, and insulin resistance have been suggested as the common pathophysiologic determinants [15–17].

Consideration of the reciprocal interaction between lung function and diabetes mellitus in clinical practice has been indicated to potentially improve outcomes as well as to reduce the healthcare burden of both respiratory and diabetic diseases [8] as well as insulin-resistant states.

Although as seen above, studies about the relation of lung function, diabetes, and MetS are plentiful, but studies about the relationship of insulin resistance and lung function are scarce. There have not been any studies evaluating lung functions in patients with insulin resistance in Turkey. The aim of the present cross-sectional study was to evaluate lung functions according to insulin resistance states in outpatients without respiratory disease admitted to internal medicine clinics in Turkey.

## Methods

### Study population

A total of 171 outpatients (mean (SD) age: 43.1 (11.9) years) admitted to internal medicine outpatient clinics at Istanbul Medeniyet University Goztepe Training and Research Hospital, Istanbul for routine check-up who gave informed consent were consecutively included to the study between January and May 2011. Active smoker or ex-smoker patients suffering from respiratory distress or diagnosed with certain concomitant diseases such as chronic obstructive respiratory disease, asthma, heart failure, chronic liver disease, chronic kidney failure, hypothyroidism, or any malignancy were excluded from the study as were diabetic patients under treatment with insulin or sulphonylureas. Patients were divided into two groups including patients with (*n* = 63, mean ± SD) age: 43.2 ± 12.5) years, 83.5 % female) or without (*n* = 108, mean ± SD) age: 43.0 ± 11.6) years, 93.5 % female) insulin resistance. The study was approved by the Istanbul Medeniyet University Goztepe Training and Research Hospital Clinical Research Ethics Committee (Protocol number and date: 8/D 28.12.2010).

Written informed consent was obtained from each subject following a detailed explanation of the objectives and protocol of the study, which was conducted in accordance with the ethical principles stated in the “Declaration of Helsinki”.

### Biochemical analysis

Blood specimens were collected after 12–16 hours of fasting. Roche Cobas 8000 analyzer was used for fasting plasma glucose (intra-assay cv % 1.7 and 0.7 for low and high concentrations respectively), triglycerides (intra-assay cv % 0.9 and 0.6 for low and high concentrations respectively), and HDL-C (intra-assay cv % 0.8 and 0.6 for low and high concentrations respectively). Beckman Coulter Unicel Dxl 800 (intra-assay cv % 5.6, 4.5, and 3.1 for normal, intermediate, and high concentrations respectively) was used for insulin assay. Primus MRDV with HPLC technique was used for HbA1c (intra-assay cv % 0.82, 0.91, and 0.46 for normal, intermediate, and high concentrations respectively; inter-assay cv % 2.91, 1.79, and 1.09 for normal, intermediate, and high concentrations respectively).

### Study parameters

Data on gender, age, anthropometric measurements, blood pressure, blood biochemistry (glycemic and lipid parameters), criteria for MetS, and lung function tests were collected in each patient with or without insulin resistance. Correlates of insulin resistance was determined via logistic regression analysis with inclusion of body mass index, waist circumference, serum levels for HbA1c, HDL cholesterol, and triglyceride along with FEV1 %, FEV1/FVC % and FEF 25–75 % predicted values as the variables.

### Anthropometric and blood pressure measurements

Weight and height were measured in light clothing without shoes. The BMI was calculated by dividing the weight by the square of the height (kg/m^2^). The waist circumference was measured over the umbilicus at the narrowest level between the costal margin and anterior superior iliac spine. Blood pressure was measured by the same person in each subject in supine position from both arms after at least 10 minutes of rest, provided that the blood pressure cuff covered about 80 % of the circumference of the upper arm with the lower edge 2.5–3 cm above the elbow.

### Insulin resistance

Insulin resistance was calculated using the homeostasis model assessment insulin resistance index (HOMA-IR) according to the following formula: fasting plasma glucose (mmol/L) × fasting serum insulin (mU/mL)/22.5 [18]. Insulin resistance was defined as HOMA-IR index ≥2.5.

### Metabolic syndrome

Definition of MetS was made based on ATPIII (adult treatment panel III) criteria [19] with consideration of MetS in the presence of 3 of 5 of the listed characteristics including abdominal obesity (waist circumference of >94 cm in males and >80 cm in females), elevated triglycerides (≥150 mg/dL, or concomitant lipid lowering treatment), reduced HDL cholesterol (<40 mg/dL in males and <50 mg/dL in females), elevated blood pressure (≥130/≥85 mm Hg), and raised fasting blood glucose (≥100 mg/dL, or concomitant diabetes mellitus).

### Lung function tests

Data on FEV1%, FVC %, FEV1/FVC %, peak expiratory flow (PEF %), forced expiratory flow (FEF 25–75 %), and forced inspiratory vital capacity (FIVC %) were collected in each subject via forced spirometry and static respiratory volume measurements performed by trained staff using Vitalograph Pneumotrac 6800 (Vitalograph Ltd., Ireland). All tests were carried out following guidelines proposed by the European Respiratory Society [20]. The theoretical values proposed by Roca et al. [21] and by the European Respiratory Society for static volumes were applied for spirometry [20]. Spirometric measurements of FVC and FEV1, percentage of FVC for predicted values (FVC_%pre_), and percentage of FEV1 for predicted values (FEV1_%pre_) were used as markers of restrictive lung dysfunction. The FEV1/FVC ratio was calculated by dividing the measured FEV1 by the measured FVC as a marker of obstructive lung dysfunction [22, 23].

The ratio of FEV1 to FVC (FEV1/FVC) was calculated, and a value ≥70 % was considered as normal. According to a modified classification of the Global Initiative for Chronic Obstructive Lung Disease (GOLD), patients were classified as having normal spirometric values (FEV1/FVC ≥70 %, FVC ≥80 %), obstructive lung dysfunction (FEV1/FVC <70 %) and restrictive lung dysfunction (FVC <80 % predicted, FEV1/FVC ≥70 %) [24].

### Statistical analysis

Statistical analysis was made using computer software NCSS 2007 version 07.1.14 SPSS version 20.0.0.1 (SPSS Inc. Chicago, IL, USA). Categorical data were analyzed using Chi-square test. Numerical data were analyzed was using Student’s *t*-test and one-way ANOVA for variables with normal distribution, while Mann–Whitney U and Kruskal–Wallis tests were used for non-normally distributed variables. Best logistic regression model for insulin resistance was selected based on lowest Akaike information criterion (AIC) and Bayesian information criterion (BIC) values. Data were expressed as “median (min-max)” and percent (%) where appropriate. A *p* < 0.05 was considered statistically significant (Additional file 1).

## Results

### Demographics and anthropometrics in study groups

Patients with and without insulin resistance were homogenous in terms of gender and age. Insulin resistance was present in 36.8 % of patients. Mean ± SD values for BMI (body mass index) (37.0 ± 5.8 vs. 33.4 ± 5.2 kg/m2, *p* < 0.001), percentage of patients with BMI of ≥35 kg/m^2^ (61.9 vs. 28.7 %, *p* < 0.001), and Mean ± SD values for waist circumference (107.0 ± 11.5 *vs*. 98.3 ± 9.8 cm, *p* < 0.001) were significantly higher in patients with than without insulin resistance (Table 1).

**Table 1:** Demographic and clinical characteristics with respect to insulin resistance

			Insulin resistance		*p* value
		Total	Absent	Present	with *vs*. without insulin resistance
		(*n* = 171)	(*n* = 108)	(*n* = 63)	
Demographics Mean (SD)					
Age (year)		43.1 (11.9)	43.0 (11.6)	43.2 (12.5)	0.925^1^
Gender			*n* (%)		
*Male*		16 (9.4)	7 (6.5)	9 (14.3)	0.091^2^
*Female*		155 (90.6)	101 (93.5)	54 (85.7)	
Anthropometrics					
Height (cm) Mean (SD)		160.0 (7.7)	159.1 (6.8)	161.7 (8.9)	0.110^b^
Weight (kg)		160.0 (7.7)	84.4 (12.7)	96.0 (16.9)	<0.001^b^
Body mass index (kg/m^2^)					
*Overall score*	*Mean* (*SD*)	34.7 (5.7)	33.4 (5.2)	37.0 (5.8)	<0.001^b^
≤*29.9*	*n* (%)	30 (17.5)	25 (23.1)	5 (7.9)	<0.001^2^
*30*-*34.9*		71 (41.5)	52 (48.1)	19 (30.2)	
≥*35*		70 (40.9)	31 (28.7)	39 (61.9)	
Waist circumference (cm)		101.5 (11.3)	98.3 (9.8)	107.0 (11.5)	<0.001^b^
Blood pressure (mmHg)			Mean (SD)		
Systolic		131.7 (18.7)	130.6 (19.3)	133.4 (17.5)	0.220^b^
Diastolic		81.0 (11.3)	80.2 (11.4)	82.4 (11.1)	0.235^b^
Glycemic parameters			Mean (SD)		
FBG (mg/dL)		108.2 (35.1)	102.2 (32.0)	118.5 (37.8)	<0.001^b^
HbA1c (%)		6.1 (1.1)	6.0 (1.0)	6.3 (1.2)	0.013^b^
Insulin (μU/ml)		10.1 (5.9)	6.9 (2.3)	15.7 (6.1)	<0.001^b^
HOMA-IR		2.7 (1.8)	1.7 (0.5)	4.5 (1.8)	<0.001^b^
Lipid parameters			Mean (SD)		
LDL cholesterol (mg/dL)		121.5 (35.3)	122.8 (35.2)	119.1 (35.6)	0.299^b^
HDL cholesterol (mg/dL)		50.2 (10.8)	52.9 (10.9)	45.6 (8.9)	<0.001^b^
Total cholesterol (mg/dL)		198.6 (41.4)	197.7 (41.2)	200.1 (42.0)	0.719^b^
Triglyceride (mg/dL)		151.6 (86.3)	127.8 (65.9)	192.4 (101.3)	<0.001^b^
Metabolic syndrome					
Present	n (%)	109 (63.7)	57 (52.8)	52 (82.5)	<0.001^2^
Diagnostic criteria (#)	Mean (SD)	3.0 (1.0)	3.0 (1.0)	4.0 (1.0)	<0.001^b^
*Waist circumference* (*Male>* *94 cm*, *Female>* *80 cm*)		171 (100.0)	108 (100.0)	63 (100.0)	-
*Blood pressure* (≥*130*/≥*85 mm Hg*)		98 (57.3)	56 (51.9)	42 (66.7)	0.059^2^
*Fasting blood glucose* (≥*100 mg*/*dL*, *or concomitant diabetes mellitus*)		80 (46.8)	41 (38.0)	39 (61.9)	0.002^2^
*HDL cholesterol* (*Male* < *40 mg*/*dL*, *Female* <*50 mg*/*dL*)		94 (55.0)	50 (46.3)	44 (69.8)	0.003^2^
*Triglycerides* (≥*150 mg*/*dL*)		76 (44.4)	37 (34.3)	39 (61.9)	<0.001^2^
Spirometric findings			Mean (SD)		
FEV1_%pre_ (%)		93.6 (11.9)	95.2 (10.9)	91.0 (13.2)	0.111^b^
FEV1/FVC_%pre_ (%)		105.3 (5.8)	106.3 (5.2)	103.5 (6.4)	0.004^b^
PEF_%pre_ (%)		88.3 (15.5)	89.6 (14.1)	86.0 (17.4)	0.281^b^
FEF 25-75_%pre_ (%)		109.2 (22.7)	112.7 (21.8)	103.1 (23.1)	0.020^b^
FVC_%pre_ (%)		89.1 (11.3)	89.7 (10.6)	88.0 (12.5)	0.587^b^
FIVC_%pre_ %		84.3 (23.2)	86.1 (24.7)	81.3 (20.6)	0.119^b^
Respiratory function			*n* (%)		
Normal		134 (78.4)	90 (83.3)	44 (69.8)	0.039^2^
Abnormal		37 (21.6)	18 (16.7)	19 (30.2)	

*HDL* high density lipoprotein, *LDL* low density lipoprotein, *FBG* fasting blood glucose, *FEV1*_%*pre*_ forced expiratory volume in the first second of expiration for predicted values, *FVC*_%*pre*_ forced vital capacity for predicted values, *PEF*_%*pre*_ peak expiratory flow for predicted values, *FEF*_%*pre*_ forced expiratory flow for predicted values; FIVC_%pre_: forced inspiratory vital capacity for predicted values

^a^Student’s *t*-test, ^2^X ^2^ test, ^b^Mann–Whitney *U* test

### Glycemic parameters in study groups

Mean ± SD levels of FBG (118.5 ± 37.8 vs. 102.2 ± 32.0 mg/dL, *p* < 0.001), HbA1c (6.3 ± 1.2 *vs*. 6.0 ± 1.0 %, *p* = 0.013), insulin (15.7 ± 6.1 vs. 6.9 ± 2.3 μU/ml, *p* < 0.001), and HOMA-IR (4.5 ± 1.8 vs. 1.7 ± 0.5, *p* < 0.001) were significantly higher in patients with than without insulin resistance (Table 1).

### Lipid parameters in study groups

Patients with insulin resistance were determined to have significantly lower levels of HDL cholesterol (45.6 ± 8.9 vs. 52.9 ± 10.9 mg/dL, *p* < 0.001) and higher levels of triglycerides (192.4 ± 101.3 vs. 127.8 ± 65.9 mg/dL, *p* < 0.001) when compared to patients without insulin resistance (Table 1).

### MetS *in study groups*

MetS was noted in a significantly higher percentage of patients with than without insulin resistance (82.5 vs. 52.8 %, *p* < 0.001) with markedly higher overall mean ± SD) number of positive diagnostic criteria (4.0 ± 1.0 vs. 3.0 ± 1.0, *p* < 0.001) and a higher percentage of patients meeting the criteria of raised blood glucose/diabetes (61.9 vs. 38.0 %, *p* = 0.002), reduced HDL cholesterol (69.8 vs. 46.3 %, *p* = 0.003), and raised triglycerides (61.9 vs. 34.3 %, *p* < 0.001) in the former group (Table 1).

### Lung function in study groups

Mean ± SD values for FEV1/FVC (103.5 ± 6.4 vs. 106.3 ± 5.2, *p* =0.004) and FEF 25–75 (103.1 ± 23.1 vs. 112.7 ± 21.8, *p* =0.020) were significantly lower in patients with than without insulin resistance. The percentage of patients with abnormal lung function was significantly higher in the insulin resistance group (30.2 vs. 16.6 %, *p* =0.039) (Table 1).

### Correlates of insulin resistance

Lung function evaluation revealed abnormal findings in one-third of our patients with insulin resistance, almost two-fold higher than the rate noted in patients without insulin resistance. Univariate analysis revealed that waist circumference (*p* < 0.001), body mass index (*p* < 0.001), triglycerides (*p* < 0.001), and HbA1c (*p* =0.038) levels were positively associated, while HDL cholesterol (*p* < 0.001), and predicted values for FEV1 % (*p* = 0.028), FEV1/FVC % (*p* = 0.003), and FEF 25–75 % (*p* = 0.009), were negatively associated with insulin resistance. Age-adjusted analysis also revealed similar findings (Table 2).

**Table 2:** Univariate logistic regression analysis for the correlates of insulin resistance

	Overall				Age-adjusted analysis			
	Odds ratio	95 % confidence interval		*p* value	Odds ratio	95 % confidence interval		*p* value
		Minimum	Maximum			Minimum	Maximum	
Gender (being female)	0.42	0.15	1.18	0.099	0.40	0.14	1.17	0.095
Age (year)	1.00	0.98	1.03	0.924	-	-	-	-
Height (cm)	1.05	1.003	1.090	0.037	1.05	1.00	1.09	0.032
Waist circumference (cm)	1.08	1.04	1.12	<0.001	1.08	1.05	1.12	<0.001
Body weight (kg)	1.06	1.03	1.08	<0.001	1.06	1.03	1.09	<0.001
Body mass index (kg/m^2^)	1.13	1.06	1.20	<0.001	1.13	1.06	1.20	<0.001
Systolic blood pressure (mmHg)	1.01	0.99	1.03	0.347	1.01	0.99	1.03	0.334
Diastolic blood pressure (mmHg)	1.02	0.99	1.05	0.218	1.02	0.99	1.05	0.218
HbA1c (%)	1.37	1.02	1.84	0.038	1.45	1.04	2.02	0.031
Fasting blood glucose (mg/dL)	1.01	1.00	1.02	0.007	1.02	1.01	1.03	0.004
Insulin (μU/ml)	2.32	1.76	3.04	<0.001	2.43	1.82	3.24	<0.001
HDL-cholesterol (mg/dL)	0.92	0.89	0.96	<0.001	0.92	0.89	0.96	<0.001
LDL-cholesterol (mg/dL)	1.00	0.99	1.01	0.510	1.00	0.99	1.01	0.504
Total cholesterol (mg/dL)	1.00	0.99	1.01	0.703	1.00	0.99	1.01	0.709
Triglyceride (mg/dL)	1.01	1.01	1.02	<0.001	1.01	1.01	1.02	<0.001
Metabolic syndrome criteria #	1.84	1.40	2.43	<0.001	2.10	1.53	2.88	<0.001
Metabolic syndrome diagnosis	4.23	1.99	8.97	<0.001	4.83	2.19	10.66	<0.001
FEF 25-75_%pre_ (%)	0.98	0.97	1.00	0.009	0.98	0.97	1.00	0.008
FEV1_%pre_ (%)	0.97	0.94	1.00	0.028	0.97	0.94	1.00	0.028
FEV1/FVC_%pre_ (%)	0.91	0.86	0.97	0.003	0.91	0.86	0.97	0.003
FIVC_%pre_ (%)	0.99	0.98	1.01	0.301	0.99	0.96	1.02	0.342
FVC_%pre_ (%)	0.99	0.96	1.01	0.342	0.99	0.98	1.01	0.307
PEF_%pre_ (%)	0.99	0.97	1.01	0.152	0.99	0.97	1.01	0.152
Abnormal lung function test	2.16	1.03	4.52	0.041	2.29	1.05	4.96	0.036

*FEV1*_%*pre*_ forced expiratory volume in the first second of expiration for predicted values, *FVC*_%*pre*_ forced vital capacity for predicted values, *PEF*_%*pre*_ peak expiratory flow for predicted values, *FEF*_%*pre*_ forced expiratory flow for predicted values *FIVC*_%*pre*_ forced inspiratory vital capacity for predicted values

Logistic regression analysis with inclusion of BMI, waist circumference, HbA1c, HDL cholesterol, and triglycerides, as well as predicted values for FEV1/FVC% and FEF 25–75 % revealed an increase in the likelihood of having insulin resistance by 1.07 times (95 % CI 1.02–1.13, *p* = 0.011) with every 1-point increase in waist circumference, 1.01 times (95 % CI 1.0–1.01, *p* = 0.032) with every 1-point increase in triglycerides, 0.93 times (95 % CI 0.89-0.98, *p* = 0.004) with every 1-point decrease in HDL cholesterol, and 0.86 times (95 % CI 0.76–0.97, *p* = 0.012) with every 1-point decrease in percentage of FEV1/FVC predicted value. Age-adjusted analysis also revealed similar findings (Table 3).

**Table 3:** Multivariate logistic regression analysis for the correlates of insulin resistance

	Overall				Age-adjusted analysis			
	Odds ratio	95 % confidence interval		*p* value	Odds ratio	95 % confidence interval		*p* value
		Minimum	Maximum			Minimum	Maximum	
Waist circumference (cm)	1.07	1.02	1.13	0.011	1.08	1.02	1.14	0.009
Body mass index (kg/m^2^)	1.03	0.94	1.13	0.541	1.03	0.93	1.14	0.552
HDL cholesterol (mg/dL)	0.93	0.89	0.98	0.004	0.93	0.89	0.97	0.002
Triglyceride (mg/dL)	1.01	1.00	1.01	0.032	1.01	1.00	1.01	0.040
HbA1c (%)	1.22	0.81	1.83	0.340	1.09	0.69	1.71	0.717
FEV1/FVC_%pre_ (%)	0.86	0.76	0.97	0.012	0.84	0.74	0.95	0.006
FEF 25-75_%pre_ (%)	1.00	0.98	1.03	0.934	1.00	0.98	1.03	0.761

*FEV1*_%*pre*_ forced expiratory volume in the first second of expiration for predicted values, *FVC*_%*pre*_ forced vital capacity for predicted values, *FEF*_%*pre*_ forced expiratory flow for predicted values

## Discussion

Our findings revealed that insulin resistance was present in 36.8 % of outpatients admitted to internal medicine clinics, with higher rates for dysglycemia and dyslipidemia and thereby a higher prevalence of MetS in patients with than without insulin resistance. Insulin resistance was positively associated with waist circumference, BMI, and serum levels of triglycerides and HBA1c, while it was negatively correlated with HDL cholesterol levels and lung function parameters including predicted values for FEV1%, FEV1/FVC%, and FEF25–75 %. Multiple logistic regression analysis revealed waist circumference and triglyceride levels as the positive determinants while HDL levels and FEV1/FVC% were negative determinants of insulin resistance.

Accordingly, our findings in a cohort of outpatients admitted to internal medicine clinics demonstrate that impaired lung function, with FEV1/FVC% in particular, can be used to predict development of insulin resistance in agreement with data from prior studies indicating that lower lung function was associated with a state of insulin resistance, both longitudinally [6] and cross-sectionally [25].

Cross-sectional analyses that investigated the relationship between lung dysfunction and dysglycemia in individuals without diabetes found conflicting results [10, 12–14]. In a past study conducted with 5346 men in Japan with no history of diabetes or lung dysfunction, it was reported that a 10-point decrease in percentage of FEV1 predicted value was associated with an increased hazard ratio of 1.21 for diabetes after adjustment for demographic factors and body mass index [10]. FEV1 and FVC were reported to be inversely associated with insulin resistance and the prevalence of type 2 diabetes in female participants older than 60 years in the British Women’s Heart and Health Study [5], while in a younger population of non-diabetic morbidly obese women, a negative correlation of HOMA-IR with FEV1, FEF25–75, and FVC was reported [15].

The diminished lung function in patients with insulin resistance as well as identification of FEV1/FVC decline as the significant determinant of increased likelihood of insulin resistance in our study population seem consistent with past studies indicating that the risk for developing diabetes was inversely related to prior lung function [26, 27], in addition to an association between low lung function and both measures of insulin resistance and type 2 diabetes [5, 13, 28].

Hence, in our study population, that insulin resistance was negatively correlated with lung function seems notable and supports the suggestion that the metabolic pathways related to insulin resistance are crucial in initiating lung abnormalities in type 2 diabetic patients [15]. Our findings indicate FEV1/FVC% to be a significant and strong risk factor for development of insulin resistance, which emphasizes the more pronounced role of FEV1% decline than FVC% decline. Hence, our findings emphasize the likelihood of insulin resistance to be causally related to obstructive rather than restrictive respiratory patterns while supporting the statement that pre-diabetes and abdominal obesity rather than diabetes are causally related to a restrictive respiratory pattern [24]. Notably, reduced baseline FVC and FEV1 were reported to be independently related to a greater risk of future development of MetS [1], while a shared pathophysiology has been suggested to underlie this association with consideration of reduced lung volumes as the potential markers of lower physical endurance in patients at risk for the development of MetS [8]. Garcia-Larsen et al. stated that reduced FVC was related to HOMA-IR in both genders, although FVC and FEV1 were negatively related to MS in the young adult study population and in men [29].

Mechanisms involved in the insulin resistant state have been considered likely to be responsible for predisposing individuals to a lower maximal attained lung function or to an early initiation of the decline in lung function [28]. The mean age of our cases was 43.1 ± 11.9 years, with smokers, ex-smokers, and patients with respiratory distress and concomitant respiratory diseases excluded from the study; the majority of our study population was female (90.6 %). In fact, the estrogen and progesterone that appear after menopause increase susceptibility to both insulin resistance and respiratory dysfunction along with the normal physiological decline in lung function after the ages of 30–35 years for most people [15, 28]. In this regard, persistence of the relationship between insulin resistance and lung function in our patients even after the age-adjusted univariate and multivariate logistic regression analyses seems notable.

Given the BMI of ≥35 kg/m^2^ in 61.9 % and presence of MetS in 82.5 % of patients with insulin resistance and abdominal obesity in all patients with MetS in the present study, our findings strongly correlate with the data from a past study in obese patients that reported a lower FEV1/FVC ratio, indicating airway obstruction, in patients with than without MetS [24] and support the demonstrated relationship between abdominal circumference and the FEV1/FVC ratio [30].

Indeed, the cellular mechanisms underlying the insulin resistant state have been suggested to explain the observed relationship between low lung function with either cardiovascular disease or all-cause mortality in many epidemiologic studies [28]. Notably, low lung function among diabetics was reported to also be an independent predictor of all-cause mortality [11]. FEV1 was reported specifically as an independent predictor of all-cause mortality and a strong risk factor for cardiovascular disease, stroke, and lung cancer [31]. Additionally, airflow obstruction as defined by an FEV1/FVC less than 0.70 has also been linked with increased coronary artery disease morbidity and mortality in large population studies [32].

Our findings revealed that impairment of lung function might be regarded as an early manifestation of insulin resistance and that determination of FEV1/FVC should serve to detect subjects at risk for developing insulin resistance, which has been accused, at least partially, for the association of increased risk of mortality from coronary heart disease (CHD) in subjects with decreased baseline ventilatory function [33]. Therefore our findings are in line with the statement that early detection of insulin resistance may lead to effective interventions aimed at primary prevention of the syndrome as well as the risk of mortality from CHD [1].

The present study has a number of limitations that should be taken into account in evaluating the results. First, this was a cross-sectional study and, therefore, a causal link between insulin resistance and impaired lung function cannot be drawn. Second, the relatively small sample size might prevent us from projecting our results to the entire population. Third, exclusion of diabetic patients under treatment with insulin or sulphonylureas may have introduced selection bias given the likelihood of systematic exclusion of subjects with the most severe insulin resistance. Nevertheless, given that lung dysfunction as part of the pre-diabetic state has not been fully elucidated [10], our findings would contribute to a comprehension of the interaction between lung function and insulin resistance in an outpatient population.

## Conclusion

Our findings in a population of outpatients admitted to internal medicine clinics without a known respiratory disorder revealed the presence of insulin resistance in 36.8 %, with higher rates for dysglycemia and dyslipidemia and thereby higher prevalence of MetS in patients with than without insulin resistance. Increases in waist circumference and triglyceride levels and decreases in HDL cholesterol and percent value for predicted FEV1/FVC were associated with increased likelihood of insulin resistance. While the exact mechanisms by which a state of insulin resistance leads to low lung function as well as the value of introducing lung function measures into an insulin resistance prediction model remain to be elucidated, our findings emphasize that FEV1/FVC% is low, thereby indicating an obstructive respiratory pattern in the interaction between lung dysfunction and insulin resistance. Based on these findings, it seems reasonable to advocate the measurement and control of lung function along with implication of programs aimed at reduction in obesity to decrease the likelihood of insulin resistance.

## Additional file

Additional file 1:
**STROBE Statement—checklist of items that should be included in reports of observational studies.** (DOC 88 kb)

## Abbreviations

CVD: Cardiovascular disease
MetS: Metabolic syndrome
HDL: High density lipoprotein
LDL: Low density lipoprotein
FBG: Fasting blood glucose
ATPIII: Adult treatment panel III
CHD: Coronary heart disease
HOMA-IR: Homeostasis model assessment insulin resistance index
BMI: Body mass index
FEV1_%pre_: Forced expiratory volume in the first second of expiration for predicted values
FVC_%pre_: Forced vital capacity for predicted values
PEF_%pre_: Peak expiratory flow for predicted values
FEF_%pre_: Forced expiratory flow for predicted values
FIVC_%pre_: Forced inspiratory vital capacity for predicted values
FVC: Forced vital capacity
FEV1: Forced expiratory volume in the first second of expiration
